# Establishment of a pediatric trigger tool based on Global Trigger Tool to identify adverse drug events of children: experience in a Chinese hospital

**DOI:** 10.1186/s12887-020-02354-9

**Published:** 2020-09-29

**Authors:** Yi Liu, Junfeng Yan, Yunfei Xie, Yuan Bian

**Affiliations:** 1 Clinical Medical College & Affiliated Hospital of Chengdu University, Chengdu, China; 2 Sichuan Academy of Medical Sciences & Sichuan Provincial People’s Hospital, Affiliated Hospital of University of Electronic Science and Technology of China, Chengdu, China

**Keywords:** Pediatric, Drug toxicity, Trigger points, Adverse drug events, Triggers

## Abstract

**Background:**

The Global Trigger Tool (GTT),which is a method using “triggers” to review medical record retrospectively to identify possible adverse events. Several studies showed that the GTT was effective. However, there were only a few localized trigger tools that had been established to detect pediatric adverse drug events (ADEs) in China. This study aimed to establish a pediatric trigger tool based on GTT, to examine the performance by detecting pediatric inpatients ADEs in a Chinese hospital (a retrospective review), and to investigate the factors associating with the occurrence of ADEs.

**Methods:**

The triggers were established by three steps including literature search, triggers extraction and revision, and experts investigation. A retrospective cohort study was conducted to detect ADEs by using 200 pediatric inpatient records of Sichuan Provincial People’s Hospital.

**Results:**

Thirty-three preliminary triggers were established, and 2 rounds of experts investigation were conducted. Finally, 33 triggers were established. In the retrospective review, the positive trigger rate was 64.0%, while the positive predictive value (PPV) was 24.9%. The occurrence of inpatients with ADEs was 20.5%. ADEs/100 admissions were 49.0. ADEs/1000 patient days were 46.89. The most common ADE categories were leukocyte disorders, skin disorders and platelet disorders. The severity of 39 ADEs was grade 1, 55 ADEs was grade 2, 4 ADEs was grade 3. The highest frequency of ADE-related drugs was antineoplastic, followed by antibacterial. The length of stay and the leukemia in the diagnosed diseases were positively correlated with ADEs.

**Conclusions:**

The 33 pediatric triggers may detect ADEs effectively, but still need to be optimized. This study may provide some references for further research in order to improve the rationality and safety of medication.

## Background

A study of Institute for Safe Medication Practice (ISMP) showed that the incidence of potential adverse drug events (ADEs) in pediatric inpatients was about three times more than adult inpatients [1]. Chinese Adverse Drug Reaction/Event Monitoring Report in 2016 showed that children’s adverse drug reaction(ADR)/ADE reports accounted for 9.9% of all ADR/ADE reports [2], indicated that Chinese children’s ADR/ADE needs more attention. In 2003, the American Institute for Health Promotion (IHI) introduced the Global Trigger Tool (GTT), and revised in 2009 [3]. GTT is an ADE detecting method which developed on medical record review. Compared with the most widely used Voluntary Reporting Notification in China, GTT has obvious initiative. In recent ten years, several studies in domestic and overseas had shown the effectiveness of GTT in detecting ADE. IHI recommends that medical institutions should make appropriate revisions based on their own characteristics [3]. At present, there are only a few localized trigger tools for detecting pediatric ADE in China. This study would like to establish a trigger tool based on GTT, which could provide a reference for the further modification of the trigger tool and more suitable for the local pediatric inpatients.

## Methods

### Aim and design

This study attempts to establish a trigger tool based on GTT to detect ADEs in pediatric inpatients, and to investigate the factors associating with the occurrence of ADEs. The trigger tool was established by three steps including literature search, triggers extraction and revision, and experts investigation. A retrospective cohort study was conducted to detect ADEs by using 200 pediatric inpatient records of Sichuan Provincial People’s Hospital.

### Literature Search

Literature search, which contains the key words such as “pediatric trigger tool” and “trigger tool”, was performed by using the literature published from January 1996 to November 2016 on PubMed (http://pubmed.cn/) and CNKI (http://www.cnki.net/). Inclusive criteria: (1) specific items with triggers;(2) application of triggers in pediatric ADE detection; (3) the trigger had detecting results. Then, this study evaluated the quality of enrolled literatures through the Combieg cross-sectional method [4].

### Triggers Extraction and Revision

Then, extracted preliminary triggers were from the included literature, and formed a review panel which included pharmacists and physicians. The panel revised preliminary triggers were based on pediatric authority guides, and added triggers through the ADR which had been reported a large number in the Chinese National Adverse Drug Reaction Monitoring System (NADRMS) from March 6, 2007 to March 6, 2017. During the revision process, pharmacists were responsible for inquiry and collation of the references, and physicians were responsible for identifying the consistency of the contents.

### Experts Investigation (Delphi Methodology)

Delphi methodology was used to conduct experts investigation in this study. Under the principles of informed consent and voluntary participation, pediatric physicians and pharmacists were selected randomly from nationally-level medical institutions. Then the suggestions on preliminary triggers were put forward by these experts, including the rationality of triggers setting and interpretation. Next, triggers were modified based on the suggestions of experts. After a few rounds, a trigger tool which was highly recognized by experts was created finally.

### Retrospective Records Review

This review was conducted in Sichuan Provincial People’s Hospital, a tertiary teaching hospital with 4,200 beds. We selected pediatric inpatient records from January 1, 2017 to September 30, 2017. Review panel also reviewed the records. The pharmacists reviewed the records primarily, and then the physicians authenticated the consensus of the pharmacists and provided answers to the questions about findings in a specific record raised by the record reviewers. The panel identified whether there existed a positive trigger according to the records, and determined whether any ADEs occurred. If there was no positive trigger but an ADE that was identified during the review process, this ADE would also need to be recorded. ADE data was collected by three ways: (1) adverse events per 1,000 patient days; (2) adverse events per 100 admissions; (3) percent of admissions with ADE [3] .

The categories of ADEs refer to WHO’s Adverse Reaction Terminology (WHO-ART) [5].

The assessment of ADE causality was based on WHO-UMC system [6]. The causality categories included: certain, probable/likely, possible, unlikely, conditional/unclassified, unassessable/unclassifiable.

The severity of ADEs was evaluated by Common Terminology Criteria for Adverse Events (CTCAE)(Version 4.0)[7]. Grade 1: Mild; asymptomatic or mild symptoms; clinical or diagnostic observations only; intervention not indicated. Grade 2: Moderate; minimal, local or noninvasive intervention indicated; limiting age-appropriate instrumental activities of daily living(ADL). Grade 3: Severe or medically significant but not immediately life-threatening; hospitalization or prolongation of hospitalization indicated; disabling; limiting self care ADL. Grade 4: Life-threatening consequences; urgent intervention indicated. Grade 5: Death related to ADE.

### Pediatric Inpatients Characteristics

In the retrospective review, a total of 200 inpatients from 200 pediatric records were identified, including 72 females and 128 males. The age ranged from 0 days to 18 years old, and the average age was 6.04 years. The inpatients were hospitalized from 2 days to 43 days, and the average hospital day was 10.45 days. The total hospital day was 2090 days. There were 1 to 6 species of diagnosed diseases per child, and the average species/record was 1.53. The most common diagnoses were pneumonia and bronchitis (57/200, 28.5%), leukemia (27/200, 13.5%), cardiovascular disease (23/200, 11.5%), sepsis and infection (20/200, 10.0%), and tonsillitis (15/200, 7.5%). The characteristics of inpatients were shown in Table 1.

**Table 1:** Pediatric Inpatients Characteristics

Characteristics		Records(%)
age	≥ 0d & ≤ 28d	10(5.0)
	>28d & ≤ 1y	27(13.5)
	>1y & ≤ 14y	145(72.5)
	>14y& ≤ 18y	18(9.0)
gender	male	128(64.0)
	female	72(36.0)
hospital day	2 ~ 7d	97(48.5)
	8 ~ 15d	64(32.0)
	>15d	39(19.5)
number of diagnosed diseases	1	130(65.0)
	2	43(21.5)
	3	16(8.0)
	4	6(3.0)
	5	2(1.0)
	6	3(1.5)
diagnoses	pneumonia/bronchitis	57(28.5)
	leukemia	27(13.5)
	cardiovascular disease(kawasaki disease, heart failure, myocarditis, etc.)	23(11.5)
	sepsis/infection	20(10.0)
	tonsillitis	15(7.5)
	others^a^	174

^a^including genital diseases such as cryptorchidism/vaginitis (13), cysts/tumors (10), urinary system diseases (8), cerebrovascular diseases (8), skin diseases such as purpura (8), cataracts and other eye diseases (7), impaired liver function (6), gastritis/enteric inflammation (6), mononucleosis (6), appendicitis (6), fracture/malformation (6), chicken chest/funnel chest (6), lymphitis (5), etc.

## Results

### Literature Search And Triggers Extraction

Forty-five literatures were retrieved through the literature search process, and 11 of them were in accordance with the inclusive criteria [8–18]. The quality level of one literature was grade B (score 5.5) [18], and the other 10 were grade A (score 6 to 7) [8–17]. The included literatures which detected ADEs in pediatric inpatients by trigger tools were from Canada, Britain, United States, Australia, Sweden, Netherlands and China. A total of 193 triggers were extracted from the included literatures, of which 115 triggers were deleted (60 triggers were repeated contents, 2 triggers were unclear descripted contents, 33 triggers had no positive trigger or the triggers were suggested deleted by authors of the included literatures because of low positive trigger rates), and 78 triggers were retained finally.

### Triggers Revision

According to authoritative guidelines, 4 of the 78 triggers had been revised: (1) Similar triggers were merged. (2) Different indicators of some triggers were set through different age groups, such as hemoglobin (Hb), blood glucose (BG), white blood cell (WBC), thyroid stimulating hormone (TSH), etc. (3) Three indicators of the biomarkers of myocardial injury were selected simultaneously: creatine kinase isoenzyme (CK-MB), CK-MB/CK and hypersensitive troponin I (hs-cTnI). (4) Some indicators were added to the biomarkers of liver injury such as R indicator. According to the reports in NADRMS, the top 5 categories of ADRs were skin system damages (rash, itching, urticaria, erythema, etc.), vomiting, leukopenia, abnormal liver function, and constipation, with a total of 73 drugs were involved. Based on the above-mentioned drugs, 4 triggers were supplemented. At last, 33 preliminary triggers were established and they would be further revised through experts investigation.

### Experts Investigation

A total of 99 experts participated in this investigation (56 pharmacists and 41 pediatricians). In the initial round, 91.8% (101/110) of the experts responded, and 41.6% (42/101) of them gave revisions. According to the opinions of experts, the triggers were modified, and the modified contents mainly include: (1) adjusting the contents of 29 triggers; (2) deleting one trigger which was lack of evidence (relieve antibiotic-related diarrhea by antidiarrheal or microecological drugs). One trigger (pediatric glucocorticoid-induced osteoporosis (GIPO) ) was added. In the second round, 98.0% (99/101) of the experts responded, and 3.0% (3/99) of them gave revised opinions, which includes: revising the contents of 2 triggers.

### Triggers

Finally, 33 pediatric triggers were established, and 3 modules include: 16 laboratory test triggers, 13 medication triggers, and 4 symptom triggers.

In the retrospective review, 128 records had at least one positive trigger. The rate of positive triggers was 64.0% (128/200), and 95% CI was 57.0%~71.0%. The triggers totally had 394 positive triggering, and 98 ADEs were identified from 41 records. The ADE positive predictive value (PPV, %) of the triggers was 24.9% (98/394), and the 95% CI was 20.1%~28.0%. Another 7 cases of ADEs involving 7 records were none positive trigger during the review process, while they were also been recorded. The triggers and PPV were shown in Table 2. The identified ADEs were shown in Table 3.

**Table 2:** The Triggers and PPV

Modules	No.	Triggers	Interpretation	Positive triggers	ADEs	PPV^a^(95%CI),%
**Laboratory test**	L1	K<3mmol·L^−1^ [8, 9, 15, 17, 18]	hypokalemic drugs used	5	3	60.0(17.1~100.0)
	L2	K>5.5mmol·L^−1^ [19]	hyperkalemic drugs used	12	4	33.3(6.7~60.0)
	L3	Na<130mmol·L^−1^ [8, 9, 13, 14, 17]	hyponatremic drugs used	4	2	50.0(1.0~99.0)
	L4	ALT≥3ULN&R≥5, ALP≥2ULN&R≤2, ALT≥3ULN,ALP≥2ULN&2<R<5 [20] R=(ALT measured value/ALTULN)/(ALP measured value/ALPULN)	hepatotoxic drugs used	9	4	44.4(11.9~76.9)
	L5	PG<2.78mmol·L^−1^(50 mg/dL) [21] PG<2. 2mmol·L^−1^(neonates) [22] PG≤3.9 mmol·L^−1^(diabetics) [23]	insulin or hypoglycemic drugs used	4	0	0^b^
	L6	PG>8.33mmol·L^−1^(150 mg/L) [16] or fasting glucose≥7.8mmol·L^−1^ [18] PG>7mmol·L^−1^(neonates) [24]	hyperglycaemic medications use or hypoglycemic drugs used inappropriately	12	10	83.3(62.~100.0)
	L7	creatinine or urea>2×baseline [9, 11, 12, 16–18]	nephrotoxic drugs used	2	2	100.0^b^
	L8	partial thromboplastin time>100 s [11, 12, 14, 16–18]	heparin used excessively	0	-^c^	-^c^
	L9	INR>3[25]	warfarin used excessively	2	1	50.0^b^
	L10	thrombocytes<50×10 ^9^ ·L^−1^ [9, 15, 18]	thrombocytopenia drugs used	16	9	56.3(31.9~80.6)
	L11	TSH≥10.0mIU·L^-1^ [26]TSH≥20mIU·L^-1^(neonates)[27]	antithyroid drugs used	1	0	0^b^
	L12	TSH<0.1mIU·L^−1^ [28]	hyperthyroidism drugs used	0	-^c^	-^c^
	L13	WBC<12×10^9^·L^−1^(neonates)[29]WBC<11×10^9^·L^−1 ^(6m~2y) WBC<5×10^9^·L^−1^(2y~14y) WBC<4×10^9^·L^−1 ^(14y~18y)	drugs which induced leukopenia used	46	21	45.7(31.3~60.1)
	L14	hs-TnI>0.0156µg·L^−1^ or CK-MB>25U·L^−1^(>15y)or CK-MB>37U·L^−1^(4y~15y) or CK-MB>45U·L^−1^(1y~3y) or CK-MB>58U·L^−1^(<1y) [30]or CK-MB/CK>5%[31](except cardiopath)	cardiotoxic drugs used	35	5	14.3(2.7~25.9)
	L15	Hb>199g·L^−1^ (neonates) [32] Hb>100g·L^−1^(2m~3m) Hb>125g·L^−1^(3m~12m) Hb>129g·L^−1 ^(1y~3y) Hb>137g·L^−1 ^(4y~8y male)Hb>131g·L^−1^(4y~8y female) [33] Hb>152g·L^−1 ^(9y~14y male) Hb>147g·L^−1^(9y~14y female) Hb>175g·L^−1^(15y~18y male) Hb>150g·L^−1^(15y~18y female) [34]	recombinant human erythropoietin used	6	0	0^b^
	L16	BMD- Z<-2.0 & clinical fracture history [35]	anti-osteoporosis drugs used	0	-^c^	-^c^
**Medication**	M1	Vitamin K given (except routine neonatal dose) [8–18]	after warfarin administration	2	0	0^b^
	M2	protamine given [11, 12, 14, 18]	after heparin administration	1	0	0^b^
	M3	heparin or low-molecular-weight heparin given [8]	after coagulant(Vitamin K) administration	7	0	0^b^
	M4	antihistamine+calglucon+glucocorticoid/adrenaline+dexamethasone given[8–12, 16–18]	after druge allergy or anaphylaxis / anaphylactic shock caused by transfusion	5	0	0^b^
	M5	narcan (naloxone) /nalmefene given [8–18]	after opioids poisoning	0	-^c^	-^c^
	M6	50% glucose injection (neonates 10%) [36] given	after drug-induced severe hypoglycemia	14	0	0^b^
	M7	Antiemetic(granisetron/ ondansetro/ tropisetron /metoclopramide)given [8–10, 13, 16–18]	after surgery,chemotherapy or drugs induced vomiting	17	2	11.8(0~27.1)
	M8	oral vancomycin or metronidazole [9, 10, 16]	after pseudomembranous colitis/staphylococcal enteritis caused by clostridium difficile due to long-term use of broad-spectrum antibiotics	8	0	0^b^
	M9	laxative or stool softener given [11–13, 17, 18]	after drug-induced constipation	8	3	37.5(4.0~71.1)
	M10	glutathione / glycyrrhizic acid preparation / polyene phosphatidylcholine given [18]	after drug-induced liver injury	31	8	25.8(10.4~41.2)
	M11	flumazenil given [11–13, 17, 18]	after severe hypotension and sedation induced by excessive benzo two nitrogenous drugs	1	1	100.0^b^
	M12	nystatin and sodium bicarbonate for external use [18]	candida infection associated with long-term use of antibiotics / hormones / immunosuppression	16	6	37.5(13.8~61.2)
	M13	intravenous bolus≥10 ml·kg^−1^ colloid or crystalloid given [9, 10]	after septic shock	75	1	1.3(0.3~3.9)
**Symptoms**	S1	rash/itching [8, 18]	after administration of antibiotics / drugs that cause skin reactions	15	11	73.3(51.0~95.7)
	S2	oversedation/hypotension/falls [16–18]	after administration of antihypertensive drugs, sedative hypnotics and other 7drugs	12	7	58.3(30.4~86.2)
	S3	admitted to ICU /rescue [18]	ADE causes serious illness	23	7	30.4(11.6~49.2)
	S4	abrupt medication stop(long-term use of anticoagulants, antihypertensives, hypolipidemic, hypoglycemic or hormones)[9, 10, 12, 15–18]	ADE causes withdrawal or ADE appears due to withdrawal	5	5	100.0^b^

*ALT* alanine aminotransferase; *ALP* alkaline phosphatase; *PG* blood glucose; *TSH* thyroid stimulating hormone; *WBC* white blood cells; *INR* international normalized ratio; *hs-TnI* hypersensitive troponin I; *CK-MB* creatine kinase isoenzymes; *CK* creatine kinase; *Hb* hemoglobin; *BMD* bone mineral density

^a^PPV=ADEs/ positive triggers.

^b^When the ADEs were close to 0,or the positive triggers were too small, the 95% CI of the PPV was not statistically significant.

^c^none

**Table 3:** The identified ADEs

Data collection methods	Identified by 33 triggers	Non positive trigger	Total
Percent of admissions with ADE(95%CI)^a^	20.5%(14.9%~26.1%)	3.5%(1.0%~6.1%)	24.0%
ADEs/100 admissions(95%CI)^b^	49.0(42.1 ~ 55.9)	3.5(1.0 ~ 6.1)	52.5
ADEs/1000 patient days(95%CI)^c^	46.89(37.8 ~ 56.0)	3.4(0.9 ~ 5.8)	50.24

*ADE* adverse drug event.

^a^Percent of admissions with ADE = Total number of records with at least one event/Total records reviewed x 100

^b^Adverse events per 100 admissions = Total number of adverse events/Total records reviewed x 100

^c^Adverse events per 1,000 patient days = Total number of adverse events/Total length of stay(LOS) for all records reviewed x 1,000

### Characteristics of ADEs

**Fig. 1:**
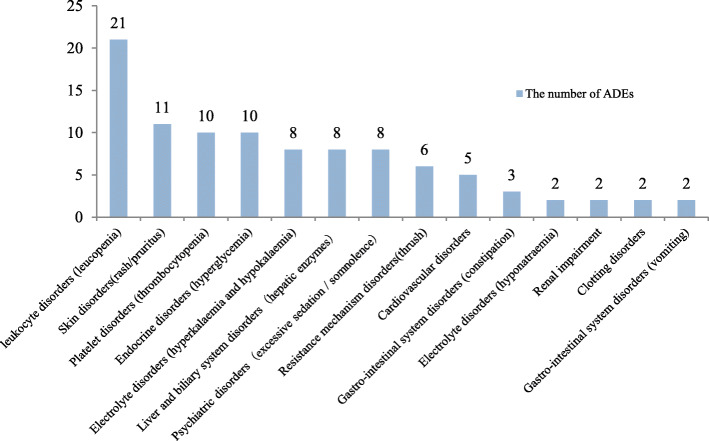
The categories of ADEs

The categories of ADEs: 98 ADEs involving 14 categories. The most common 3 categories were leukocyte disorders (21/98, 21.4%), skin disorders (11/98, 11.2%) and platelet disorders (10/98, 10.2%). The categories of ADEs were shown in Fig. 1.

**Fig. 2:**
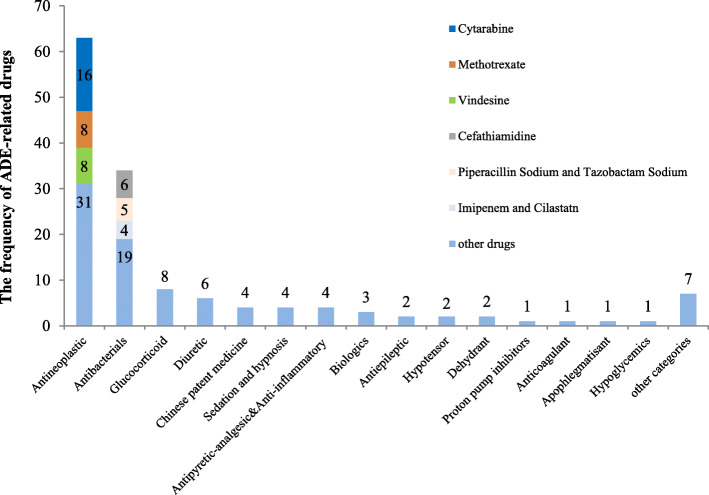
The categories of ADE-related drugs

The assessment of ADE causality: according to the WHO-UMC method, 4 cases (4.1%) were assessed as certain, 73 cases (74.5%) were assessed as probable/likely, 19 cases (19.4%) were assessed as possible, 1 case (1.0%) was assessed as conditional/unclassified due to the incomplete information of records, and 1 case (1.0%) was assessed as conditional/unclassified due to the contradiction between physician record and nursing record. The ADE-related drugs: a total of 52 drugs involving 16 categories. Antineoplastics had the highest frequency of occurrence, accounting for 44.1% (63/143), the top 3 drugs were cytarabine, methotrexate and vindesine; followed by antibiotics, accounting for 23.8% (34/143), the top 3 drugs were cefathiamiadine, piperacillin sodium and tazobactam sodium, imipenem and cilastatn. The ADE-related drugs were shown in Fig. 2.

The severity grade of ADEs: 39 ADEs were grade 1 (39/98, 39.8%), 55 ADEs (55/98, 56.1%) were grade 2 and 4 ADEs were grade 3 (4/98, 4.1%).

### Risk Factors Associated With the Occurrence of ADEs

A regression model was established. After 2 rounds of variable screening, the “length of hospital day” and “leukemia” were included in the regression model. For each additional day of hospital stay, the risk of ADE would be increased by 20.8%. Leukemia might be the only diagnosed disease associated with the occurrence of ADE, which was 6.8 times higher than the other diagnosed diseases. There was no significant correlation between the “gender”, “age”, “number of diagnosed disease”, “other diagnosis” and the occurrence of ADE (*P* > .05). The logistic regression model was shown in Table 4.

**Table 4:** Logistic regression model: risk factors for the occurrence of ADEs

Factors		Regression coefficient	SE	Wald χ^2^	F	OR(95% CI)	*P*
1	Length of hospital day	0.189	0.035	28.777	1	1.208(1.127 ~ 1.294)	< 0.001
2	Diagnosis ^a^: leukemia	1.929	0.588	1.573	1	6.881(2.173 ~ 21.795)	0.001

*ADE* adverse drug event.

^a^ The table only shows the diagnostic category of significant *P* < .05.

## Discussion

In this study, 33 triggers were established based on GTT and modified through three steps (literature search, triggers extraction and revision, experts investigation). The application of triggers was based on GTT in the world, and combined with the opinions of Chinese pediatric experts, the established triggers were more suitable for local needs.

Based on the recommendations of the IHI white paper, a review panel was formed to discuss each review. The pharmacists were mainly responsible for the inquiry and collation of the materials, and the physicians were mainly responsible for identifying the consistency of the query contents.

In this study, the number of ADE identified by the trigger tool (49.0ADEs/100 admissions) was significantly more than the voluntary reporting system. The data in NADRMS showed that, in Sichuan Provincial People’s Hospital, only 2 cases of pediatric ADR/ADE were reported from January 1 to September 30, 2017. The rate of positive triggers, PPV and ADE detection rate in our research, were higher than many previous studies which used trigger tools to detect pediatric ADE[8–18, 37].

The triggers should be modified to reduce the false positive and increase the accuracy. The positive trigger frequencies of M7 and M13 were higher (75 and 17 cases), while the detections of ADEs were low (1 and 2 cases), and the PPV of them were also low (1.33 and 11.76). Among M13 positive trigger patients, 45.3% (34/75) were surgical patients who were often injected with crystalloid or colloid fluid to supplement functional extracellular fluid and to regulate electrolytes. So the routine rehydration of surgical patients should be considered to be excluded from M13 trigger. Among M7 positive trigger patients, 82.3% (14/17) were leukemia children during chemotherapy. Tumor patients often use antiemetics as a preventive medication during chemotherapy in clinical. So the M7 trigger should be considered to be revised: “vomit (due to surgery or drugs)”. During the retrospective review, 7 ADEs without positive trigger had been found. Among them, 5 ADEs were “diarrhea caused by the use of broad-spectrum antibiotics”, and all the 5 patients were infected by Gram-positive cocci. The PPV of M8 trigger was 0.0% (0/8). It could be supplemented as: “oral vancomycin /metronidazole or clostridium difficile-positive”. The other 2 ADEs were “methotrexate blood concentration 24 h > 10 µmol ·L^− 1^, and rescued by calcium leucovorin”. A trigger was considered to be added to the tool: “methotrexate blood concentration abnormal and rescued by calcium folinate (after methotrexate administration).”

There were 4 triggers without positive trigger including L16 and L8, L12, M5 which were originated from previous studies in other countries. It might be caused by the following factors: (1) the sample size was not enough, (2) the 200 medical records extracted in this study were less involved in the above triggers or the related drugs were less used in the hospital. For example, in M5 trigger, opioids were often used to moderate and severe pain such as cancer. However, in recent years, antitussive drugs containing opioids were not recommended for children under 18. So in our hospital, there might be fewer children using opioids. L16 trigger was a new item added based on expert opinions, and had no positive trigger in this study. For one thing, it might be because the children didn’t measure bone density as a regular item; for another, China had always attached great importance to the rational use of glucocorticoids in children.

This study also had some limitations. It was a retrospective study of randomly selected medical records, and each patient had only one hospitalization record. We didn’t have any other records about “rechallenge”, so most ADEs could be assessed as “probable/likely”, but not “certain”, according to the WHO-UMC method. The 33 pediatric triggers established in this study were only used in Sichuan Provincial People’s Hospital. Although it was the largest hospital in Sichuan Province, we recommend conducting retrospective records review in more hospitals and the triggers would been modified in future studies.

## Conclusions

This was one of the few studies that have looked into detect ADEs in pediatric (including neonates) through establishing a trigger tool based on GTT in China. The study established 33 pediatric triggers through literature search, triggers extraction and revision, and experts investigation, to detected ADEs by retrospective cohort analysis. Our results showed that more than three-fifths pediatric inpatients had at least one positive triggers, more than one-fifth pediatric inpatients had ADEs, and the length of hospital stay and the leukemia patients were the high risk factors of ADEs. The 33 pediatric triggers may detect ADEs effectively, but still need to be optimized. This study may provide some references for further research in order to improve the rationality and safety of medication.

## Data Availability

Datasets used and/or analyzed during the current study are available from the corresponding author on reasonable request.

## Abbreviations

ADE: Adverse drug event
ADR: Adverse drug reaction
GTT: Global Trigger Tool
PPV: Positive predictive value
CTCAE: Common Terminology Criteria for Adverse Events
